# Do the Mandibular Condyles Change Their Positions within Glenoid Fossae after Occlusal Splint Therapy Combined with Physiotherapy in Patients Diagnosed with Temporomandibular Joint Disorders? A Prospective Case Control Study

**DOI:** 10.3390/jpm12020254

**Published:** 2022-02-10

**Authors:** Marcin Derwich, Elzbieta Pawlowska

**Affiliations:** 1 ORTODENT, Specialist Orthodontic Private Practice in Grudziadz, 86-300 Grudziadz, Poland; 2 Department of Orthodontics, Medical University of Lodz, 90-419 Lodz, Poland; elzbieta.pawlowska@umed.lodz.pl

**Keywords:** temporomandibular joint disorders, temporomandibular joints, occlusal splint, physiotherapy, CBCT, cone-beam computed tomography, condylar ratio

## Abstract

The research question was: do the mandibular condyles change their position within glenoid fossae after treatment combining occlusal splint therapy and physiotherapy in patients diagnosed with temporomandibular disorders (TMD)? Forty patients with TMD were included into the study. They underwent initial physiotherapy, and a six-month treatment of occlusal splint therapy with physiotherapy. Cone-beam computed tomography images of temporomandibular joints (TMJs) were taken before and after the treatment. The control group consisted of 15 asymptomatic patients, who did not receive any type of occlusal treatment. The changes in the dimension of anterior, superior, posterior, and medial joint spaces after the end of the treatment in patients with TMD were statistically insignificant. The average value of condylar ratio was significantly higher after the end of the treatment (*p* = 0.025). The changes in the condylar sagittal position were statistically insignificant. Occlusal splint therapy with physiotherapy did not change significantly the dimension of joint spaces, nor placed the mandibular condyles into the centric relation. Treatment of patients with TMD should not aim at gnathological concept of placing the mandibular condyles into centric relation, because centric relation appears not to be mandatory to achieve successful results of treatment in patients with TMD.

## 1. Introduction

The definition of centric relation (CR) is very controversial and has been changed so far over 26 times [1,2]. The recent definition of CR has been explained in the Glossary of Prosthodontic Terms Ninth Edition as a physiologic, maxillomandibular relationship, which does not depend on tooth contact, in which the mandibular condyles are in the anterior-superior position in the glenoid fossae, the mandible moves in pure rotation, and from this position different lateral, vertical, and protrusive movements can be made by the patient [3]. Moreover, CR is considered to be a reference position of high clinical usefulness and repeatability [3]. The current definition of CR does not include the position of the articular disc.

It has been hypothesized by some of the authors that occlusion may play a significant role in the development of the temporomandibular joint disorders (TMD) [4,5]. Roth noticed that TMD may be caused by mandibular deviation from CR with the concurrent presence of balancing interferences. He observed that severity and location of TMD symptoms are associated with the balancing interferences [4]. Moreover, Ikeda [6] stated that the most stable position of the temporomandibular joints (TMJs) is diagnosed when both of the condyles are in CR position with the articular discs in place and at the same time the occlusal contacts between the dental arches are bilaterally equal in maximum intercuspation.

There are several different methods to obtain CR, including: leaf gauge, tongue tip to soft palate, gothic arch tracing, myo-monitor, Roth power bite, chin-point guidance, bimanual manipulation, long-term deprogrammer with passive muscle contraction [7,8]. Bimanual manipulation, chin-point guidance, and Roth power bite appeared to have the same accuracy and reproducibility in healthy volunteers with Angle class I occlusal relationship [9]. Zonnenberg et al. [10] compared the efficacy of chin-point guidance and leaf gauge in healthy individuals and in patients diagnosed with TMD. Although, the accuracy of both methods in both groups was similar after the end of the splint treatment, the results of chin-point guidance registration in TMD patients differed from the results obtained with the leaf gauge before the onset of the splint therapy.

Occlusal splints are the long-term deprogrammers. They are considered to be one of the most accurate methods of obtaining CR, because they efficiently deprogram muscle memory [7,8]. Long-term deprogramming allows the musculature to position the condyles into CR [8]. One of the major advantages of occlusal splints as deprogrammers is the fact that occlusal splints are patient-determined, which means the force to sit the condyles within the glenoid fossae originates from patients’ muscles and not from the force applied by the physician [8]. Occlusal splints are used in the noninvasive treatment of patients diagnosed with TMD [11,12,13]. The clinical efficacy of occlusal splints seems to be controversial. Although, some of the authors emphasize the positive effects of the occlusal splints on TMD pain reduction, as well as on increasing maximum mouth opening [12,13,14], according to the other authors the evidence that confirms the efficacy of occlusal splints is of either low or very low quality and further studies are needed [15,16].

Physiotherapy is another noninvasive method of treatment of patients with TMD [11,13]. Manual therapy has been found effective in the treatment of TMD [17,18]. It can be performed either alone or in combination with occlusal splint therapy.

Although, the effects of occlusal splint therapy and physiotherapy have been discussed independently in terms of pain reduction and maximum mouth opening, nothing is known about the effects of occlusal splint therapy and physiotherapy combined together on the condylar position within the glenoid fossa. Therefore, the research question was: do the mandibular condyles move to the anterior-superior position in the glenoid fossae after the treatment combining occlusal splint therapy and physiotherapy in patients diagnosed with TMD?

The null hypothesis was that the sagittal and vertical positions of mandibular condyles within the articular fossae do not change in patients diagnosed with TMD after the end of occlusal splint therapy combined with physiotherapy.

## 2. Materials and Methods

This prospective, case control study was approved by the Medical Board Ethical Committee of Regional Medical Chamber in Gdansk, Poland (KB-17/21) and was conducted with the ethical principles of the World Medical Association Declaration of Helsinki. Informed consent was signed and obtained from all of the participants.

### 2.1. Participants

All of the participants from the study group suffered from TMD and underwent initial series of physiotherapy and six months of combined treatment, including occlusal splint therapy and physiotherapy. The diagnosis of TMD was made on the basis of Diagnostic Criteria for Temporomandibular Disorders (DC/TMD).

There were generally healthy patients with asymptomatic TMJs included into the control group. Those patients underwent the process of orthodontic diagnosis twice, including imaging of TMJs, because due to the financial reasons, they did not start the orthodontic treatment immediately after the initial treatment plan had been presented, but returned for the second diagnosis from 1 to 2 years later. Patients from the control group did not undergo any type of dental treatment (including: conservative dentistry, prosthodontics, orthodontics), occlusal splint therapy, nor physiotherapy.

Cone-beam computed tomography (CBCT) images of temporomandibular joints (TMJs) were taken before the onset and after the end of the treatment in patients diagnosed with TMD. The CBCT images were blinded by one of the researchers (EP) so that the second researcher (MD) did not know, to whom the particular CBCT examination belonged and whether it had been taken before or after the end of the treatment. The study was performed in the specialist orthodontic private practice in Grudziadz (Poland). The list of inclusion and exclusion criteria to this study is presented in Table 1.

### 2.2. Intervention

Anamnesis, extraoral, and intraoral examinations, as well as CBCT images of TMJs were performed by one of the researchers (MD). There were examined: head and neck muscles, TMJs, and occlusion. The pain level in the area of TMJs was assessed by the patients on the basis of 4-grade scale (0—no pain, 1—light pain, 2—moderate pain, 3—severe pain). Patients were asked to assess pain in the area of their TMJs twice: before and after the end of the therapy. To measure the maximum mouth opening a digital caliper was used. The measurements were performed intraorally as a distance between incisal edges of upper incisors and incisal edges of lower incisors in maximum opening. Maximum mouth opening was measured twice: before and after the end of the therapy.

CBCT images of TMJs were taken on MyRay Hyperion X9 3D (CEFLA, Imola, Italy). The parameters of exposition were: 90 kV, 18 mAs, exposition time of 3.6 s, field of view (FOV) was 8 × 5 cm, and the thickness of slices was 0.3 mm. While the CBCT examination was being taken, the patients were sitting on the wooden chair, holding their heads straight, looking into the mirror’s reflection of their eyes, with the teeth closed in position of maximum intercuspation. The CBCT examination was performed with the ALARA principle (As Low As Reasonably Achievable).

Patients qualified for the study started their treatment with 6 weeks of head and neck physiotherapy (appointments with physiotherapists took place once per week). Furthermore, all of the patients were given exercises to be performed at home, including Rocabado’s 6x6 exercises [19]. At the end of the initial physiotherapy session, patients underwent bite registration with Roth power bite technique (bite du jour). On the basis of the bite registration, the hard, acrylic, maxillary occlusal splints with anterior and bilateral canine guidance were manufactured. Patients were told to wear occlusal splints day and night for 6 months. Occlusal splints could have been taken off only for cleaning and brushing teeth. The occlusal splints were adjusted at every appointment so that all lower teeth had at least one contact point with the splint in static position. When the participant was moving the mandible forward, only lower central incisors were touching the splint (incisor guidance) and the remaining teeth stayed apart from the splint. Finally, when the patient was moving the mandible laterally, only the ipsilateral canine was touching the splint (canine guidance). All of the balancing contacts were removed from the splint. There were 12 check-up appointments with the occlusal splint throughout the 6-month treatment period (4 times once a week, 4 times once every second week, and 4 times every third week). During each check-up appointment, the splint was equilibrated as previously described. Participants attended physiotherapy sessions before every second check-up with occlusal splint. After the end of the treatment, the CBCT images of TMJs were taken once again with the same protocol as described above.

### 2.3. Outcome Measures

The primary outcomes were: to measure the sagittal and vertical position of the mandibular condyles within the glenoid fossae after occlusal splint therapy combined with physiotherapy in patients diagnosed with TMD. The secondary outcome was to assess the changes within the distance between the mandibular condyles and the medial wall of glenoid fossae after occlusal splint therapy combined with physiotherapy in patients diagnosed with TMD.

The iRYS Software version 6.2 (CEFLA, Imola, Italy) was used to perform all of the measurements in the CBCT images. All of the measurements were performed in the 0.3-mm thickness axial and sagittal slices of the mandibular condyle. The axial slice with the largest mediolateral dimension of the mandibular condyle was selected for further measurements. The position of the coronal axis was adjusted so that it was covering the line connecting the most prominent points on medial and lateral poles of the mandibular condyle. The position of the sagittal axis was adjusted so that it was perpendicular to the coronal axis and at the same time was crossing in the middle the distance between the most prominent points on medial and lateral poles of the mandibular condyle. The obtained sagittal view was the second slice selected for the measurements.

Table 2 presents the list of points and lines used to analyze the position of mandibular condyle within the glenoid fossa.

**Figure 1:**
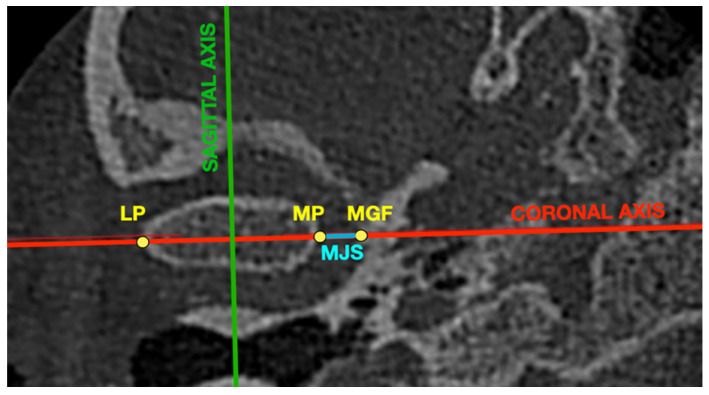
Axial view of the mandibular condyle with marked points and lines presented in Table 2. LP—lateral pole of mandibular condyle, MGF—medial wall of glenoid fossa, MJS—medial joint space, MP—medial pole of mandibular condyle.

Figure 1 presents the axial view of the mandibular condyle with marked points and lines presented in Table 2.

**Figure 2:**
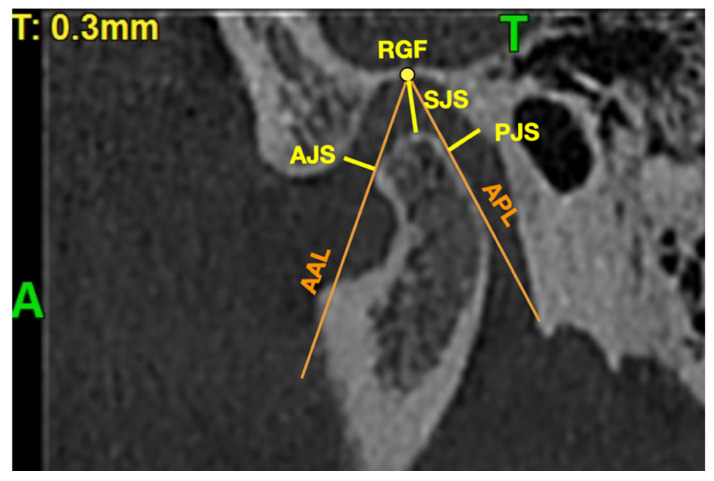
Sagittal view of the mandibular condyle with marked points and lines presented in Table 2. A—anterior, AAL—auxiliary anterior line, AJS—anterior joint space, APL—auxiliary posterior line, PJS—posterior joint space, SJS—superior joint space, RGF—roof of glenoid fossa, T—top.

Figure 2 presents the sagittal view of the mandibular condyle with marked points and lines presented in Table 2.

The sagittal position of the mandibular condyle within the glenoid fossa was assessed on the basis of the formula presented by Pullinger and Hollender [20]:

(1) $$\mathrm{condylar}\;\mathrm{ratio}=\frac{P-A}{P+A}\times 100\%$$

where: P—posterior joint space and A—anterior joint space.

Table 3 presents interpretation of the Pullinger and Hollender’s formula on the basis of the literature [20].

The treatment effects have been analyzed on the basis of the condylar ratio changes. The condylar ratio changes of 5% or more have been arbitrarily accepted as a success. The decrease of condylar ratio of 5% or more was regarded as a negative result. The changes of condylar ratio smaller than 5% (either increase or decrease) were considered to be neutral result.

ΔC_on_R_at_ (%) = C_on_R_at after treatment_ (%) − C_on_R_at before treatment_ (%), (2)

where: C_on_R_at_—condylar ratio.

### 2.4. Statistical Analysis

All statistical analyses were performed with Statistica 13.0 software (Dell Inc., Aliso Viejo, CA, USA). There were calculated: mean differences, standard deviations, medians, upper and lower quartiles, and ranges. To check whether the differences before and after the end of the treatment were statistically significant the below listed tests were applied: *t*-Student test, and U-Mann–Whitney, Pearson chi-square test. To assess the connection between the measured parameters, the correlations were analyzed. The statistical significance level was set at *p* = 0.05.

## 3. Results

### 3.1. Flow of Participants

There were 44 patients diagnosed with TMD included into the study. A total of 40 patients (32 women and 8 men) completed the six-month protocol of treatment. Four patients were excluded from the study during the first month, because they did not wear the occlusal splint as it had been recommended and finally they declined to participate. The average age of the participants from the study group was 28.4 ± 10.2 years old (median: 27 years). There were: 29 patients (72.5%) diagnosed with myalgia, 11 patients (27.5%) diagnosed with arthralgia, 13 patients (32.5%) diagnosed with headache attributed to TMD, 31 patients (77.5%) diagnosed with disc displacement with reduction, 3 patients (7.5%) diagnosed with disc displacement with reduction with intermittent locking, 5 patients (12.5%) with degenerative joint disease, and finally 11 patients (27.5%) diagnosed with subluxation. 

The control group consisted of 15 people (12 women and 3 men) with the average age of 31.3 ± 12.9 years old. Both of the groups did not differ significantly regarding the average age and the distribution of sex among the participants. Table 4 presents the comparison of age and sex between the examined groups.

**Figure 3:**
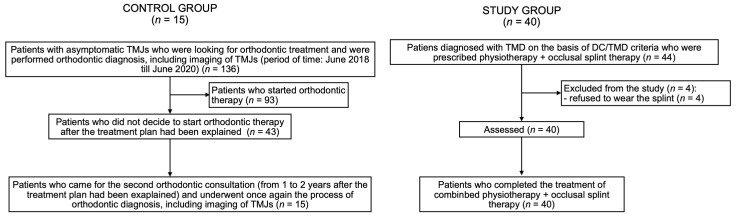
Flow of the participants during the study. DC/TMD—diagnostic criteria for temporomandibular joint disorders; TMJ—temporomandibular joint; TMD—temporomandibular joint disorders.

Figure 3 presents flow of the participants during the study.

### 3.2. Symptoms of TMD within the Study Group

Having completed a six-month period of treatment, patients from the study group reported significant decrease of pain in the area of TMJs (*p* < 0.0001). Moreover, the maximum mouth opening significantly increased after the end of the therapy (*p* = 0.0011). Table 5 presents the average values of maximum mouth opening and pain scores before and after the end of the treatment.

### 3.3. Position of Mandibular Condyles

**Figure 4:**
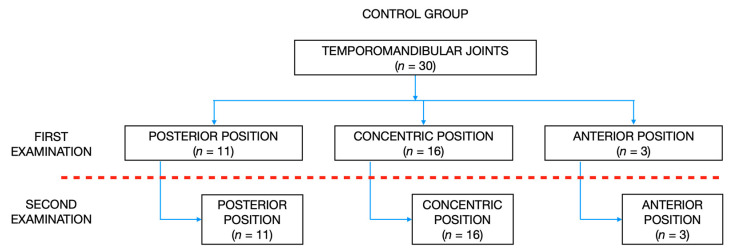
The distribution of condylar sagittal positions within the glenoid fossae in the control group during initial and second examination.

According to the Pullinger and Hollender’s formula, there were: 11 TMJs posteriorly positioned, 16 TMJs concentrically positioned, and 3 TMJs anteriorly positioned in the control group during the initial examination. Second examination, which was performed over one year after the initial examination, revealed no changes in the sagittal position of condyles within the glenoid fossae. Figure 4 presents the distribution of condylar sagittal positions within the glenoid fossae in the control group during initial and second examination.

There were no statistically significant differences between the initial and second examination performed in the control group regarding the dimension of anterior, posterior, superior, and medial joint spaces.

Table 6 presents the average values of different joint spaces and condylar ratio during initial and second examination in the control group.

Contrary to the control group, there were: 42 TMJs posteriorly positioned, 27 TMJs concentrically positioned, and 11 TMJs anteriorly positioned within the glenoid fossa in the study group before the onset of the treatment. At the end of the treatment, there were: 39 TMJs posteriorly positioned, 23 TMJs concentrically positioned, and 18 TMJs anteriorly positioned. However, the changes in the distribution of condylar sagittal positions before and after the end of the treatment were statistically insignificant (*p* = 0.346). 

**Figure 5:**
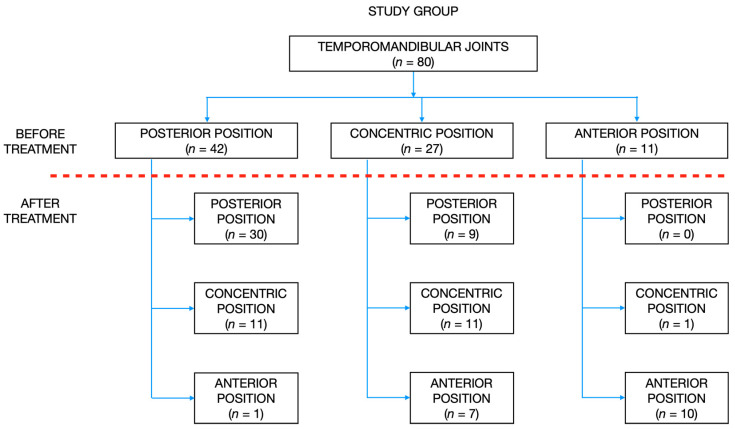
The distribution of condylar sagittal positions within the glenoid fossae in the study group before and after the treatment.

Figure 5 presents the distribution of condylar sagittal positions within the glenoid fossae in the study group before and after the treatment.

There were no statistically significant differences before and after the end of the treatment regarding the dimension of anterior, posterior, superior, and medial joint spaces. The average value of condylar ratio was significantly higher after the end of the treatment (*p* = 0.025). The number of condyles anteriorly positioned increased form 11 (13.7%) before the onset of the treatment to 18 (22.5%) after the end of the treatment. However, this increase was statistically insignificant.

Table 7 presents the average values of different joint spaces and condylar ratio before and after the end of the treatment in the study group.

**Figure 6:**
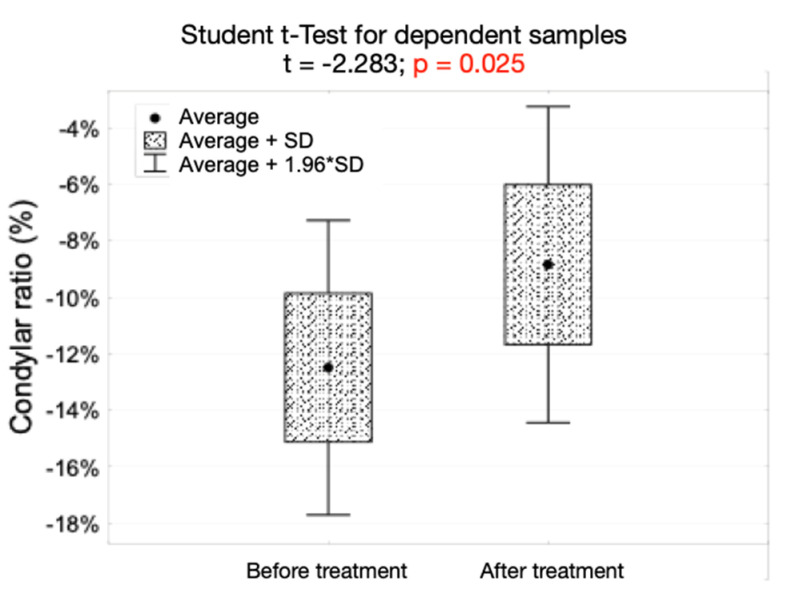
The values of condylar ratio before and after the end of the treatment, as well as the results of Student *t*-test for dependent samples in the study group.

Figure 6 presents the values of condylar ratio before and after the end of the treatment, as well as the results of Student *t*-test for dependent samples in the study group. 

There were no statistically significant differences between right and left TMJs regarding the changes in the dimension of each joint space that occurred throughout the treatment. 

Table 8 presents the average changes in the values of different joint spaces after the end of the treatment in right and left TMJs in the study group.

**Figure 7:**
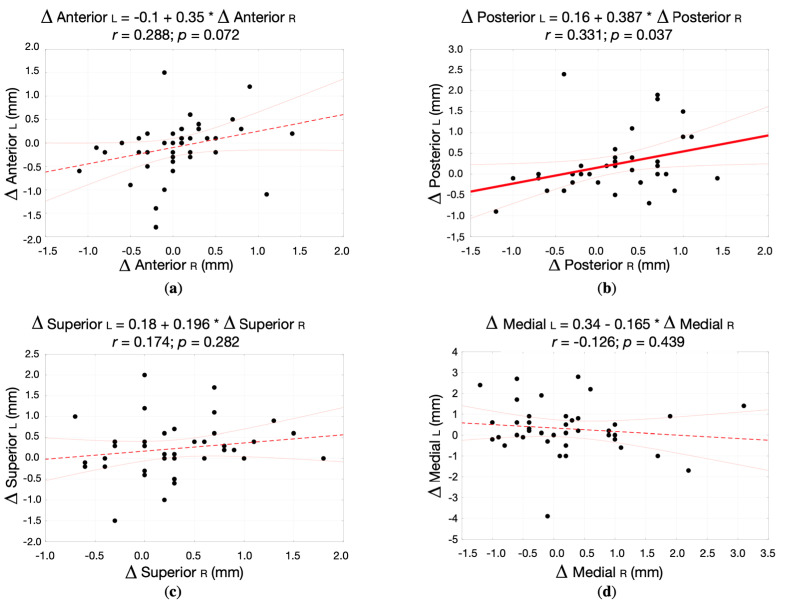
The correlation between the changes in the dimension of different joint spaces within right and left TMJs (correlation diagrams, correlation coefficient, and regression straight line equation) in the study group: (**a**) Anterior joint space; (**b**) Posterior joint space; (**c**) Superior joint space; (**d**) Medial joint space.

Figure 7 presents the correlation between the changes in the dimension of anterior, posterior, superior, and medial joint spaces within right and left TMJs (correlation diagrams, correlation coefficient, and regression straight line equation) in the study group.

The average changes in the dimension of the anterior, superior, and medial joint spaces that occurred throughout the treatment in right and left TMJs did not correlate with each other. There was only weak, positive correlation (r = 0.331, *p* = 0.037) regarding the changes in the dimension of the posterior joint space between right and left TMJs in the study group.

**Figure 8:**
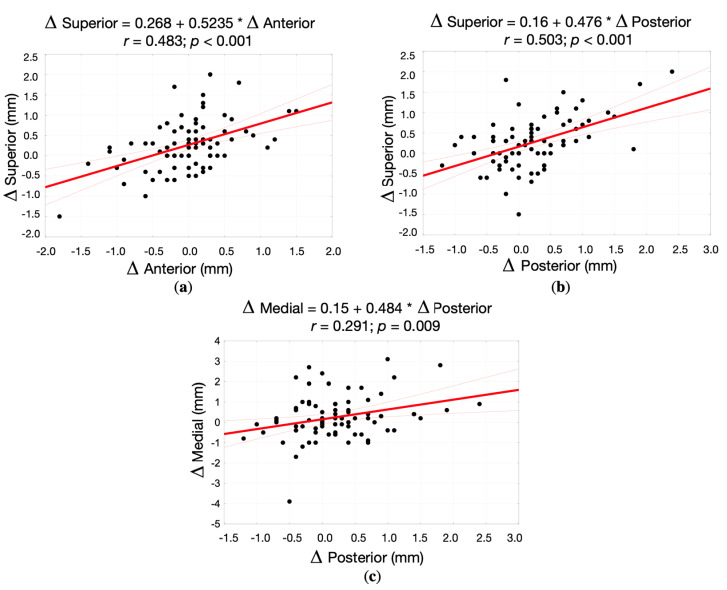
The correlation between the changes in the dimension of different joint spaces in all (right and left) TMJs (correlation diagrams, correlation coefficient, and regression straight line equation) in the study group: (**a**) Superior vs. Anterior joint spaces; (**b**) Superior vs. Posterior joint spaces; (**c**) Medial vs. Posterior joint spaces.

Having analyzed the changes of the joint spaces in all (right and left) TMJs in the study group, there were three statistically significant positive correlations between the changes in the dimension of the superior and anterior joint spaces (r = 0.483; *p* < 0.001), superior and posterior joint spaces (r = 0.503; *p* < 0.001), medial and posterior joint spaces (r = 0.291; *p* = 0.009). Figure 8 presents the correlation between the changes in the dimension of anterior, posterior, superior, and medial joint spaces in all TMJs (correlation diagrams, correlation coefficient, and regression straight line equation).

There were no statistically significant differences between right and left TMJs regarding the obtained results of the treatment in the study group. Table 9 presents the results of the treatment assessed on the basis of the of the condylar ratio changes in the study group.

The increase of condylar ratio of 5% or more was obtained in 46.3% of TMJs in the study group. There were eight patients (20%) in the study group with asymmetrical changes of the condylar sagittal position within the TMJs, namely: three patients with the increase of condylar ratio in right TMJ and decrease of condylar ratio in left TMJ, and five patients with the decrease of condylar ratio in right TMJ and increase of condylar ratio in left TMJ.

Table 10 presents the comparison of the average changes in the values of different joint spaces and the average changes in condylar ratio between the examined groups. Only the average changes regarding the dimension of the superior joint space differed significantly between the groups (*p* = 0.018). 

### 3.4. Predictive Factors for Obtaining Anterior Condylar Position after the End of the Treatment 

Receiver operating characteristic (ROC) curves analysis was used to assess sensitivity and specificity of different predictive factors for obtaining anterior condylar position after the end of the treatment. Table 11 presents the results of the ROC curves analysis.

Table 12 presents predictive factors for obtaining anterior condylar position after the end of the treatment.

There were several predictive factors for obtaining anterior condylar position after the end of the treatment, namely: C_on_R_at_ _before treatment_ ≥ 0.0%, anterior joint space dimension before treatment less than 2.1 mm, and posterior joint space dimension before treatment more than 2.4 mm.

Table 13 presents the results of the univariate and multivariate logistic regression analyses.

According to the multivariate logistic regression analysis, there are two independent predictors of obtaining C_on_R_at_ > 12% after the end of the treatment, namely: C_on_R_at_ before treatment of 0.0% or more, and anterior joint space dimension before treatment less than 2.1 mm. The probability of obtaining C_on_R_at_ > 12% after the end of the treatment can be calculated with the below presented equation:

(3) $$P(Y=1|x_{1},x_{2})=\frac{\exp(-3.62+1.67\cdot x_{1}+2.97\cdot x_{2})}{1+\exp(-3.62+1.67\cdot x_{1}+2.97\cdot x_{2})}$$

where: 

P(Y = 1|x_1_, x_2_)—probability of obtaining C_on_R_at_ > 12% after the end of the treatment

x_1_—anterior joint space before treatment < 2.1 mm (yes: 1, no: 0)

x_2_—C_on_R_at_ before treatment ≥ 0.0% (yes: 1, no: 0).

## 4. Discussion

According to our knowledge, this is the first study that prospectively analyzed the changes of the mandibular condyles’ positions within the glenoid fossae in patients diagnosed with TMD who had been treated with occlusal splint therapy combined with physiotherapy.

Physiotherapy and occlusal splint therapy appeared to be effective in pain reduction, as well as in increasing maximum mouth opening in patients diagnosed with TMD. This observation was also confirmed in other studies [21,22].

Having analyzed the CBCT images to assess the position of mandibular condyles within glenoid fossae, we did not observe any significant changes in the dimension of anterior, superior, posterior, and medial joint spaces after the end of the treatment in patients diagnosed with TMD. Moreover, there were no statistically significant differences between right and left TMJs regarding the changes in the dimension of each joint space that occurred throughout the treatment. 

The condylar ratio, assessed on the basis of the Pullinger and Hollender’s formula [20], significantly increased after the end of the treatment (*p* = 0.025). This could have been caused by muscle deprogramming and subsequently either forward movement of mandibular condyles within the glenoid fossae, clockwise rotation of mandibular condyles within the glenoid fossae (this is related to the anatomy of the mandibular condyles [23], because during clockwise rotation of mandibular condyles, the most superior part of the condylar head moves forward, and the anterior joint space decreases), or by combination of the two mentioned above. Clockwise rotation of mandibular condyles was caused by the increased intraoral vertical dimension because of the occlusal splint worn day and night. This may be speculated to be the major effect of occlusal splints on condylar three-dimensional position within the glenoid fossae, because the changes in the distribution of condylar sagittal positions (posterior, concentric, anterior) within glenoid fossae, were statistically insignificant. Although, 19 out of 80 TMJs moved forward after the end of the treatment, only eight TMJs moved to the anterior position. Therefore, these results do not confirm the thesis that after occlusal splint therapy, mandibular condyles will move to centric relation, described as the most superior and anterior part of glenoid fossa. Moreover, ten TMJs moved distally after the end of the treatment (nine concentric TMJs moved to the posterior position and one anteriorly positioned TMJ moved to the concentric position). The vast majority of TMJs (51 TMJs, 63.75%) did not change their initial sagittal position within the glenoid fossae after the end of the therapy.

To monitor the efficacy of the prescribed therapy in changing the sagittal condylar positions within the glenoid fossae to the more anterior ones, we arbitrarily accepted the condylar ratio changes of 5% or more as a success, whereas the decrease of condylar ratio of 5% or more was regarded as a negative result. According to our study, more than half of the examined TMJs (43 TMJs, 53.75%) did not achieve improvement of the value of condylar ratio of at least 5%. Moreover, although there were no statistically significant differences regarding the effects of treatment between right and left TMJs, there were eight patients who presented asymmetrical changes of the condylar sagittal position within the glenoid fossae. Taking all of these measurements into consideration, it can be stated once again, that occlusal splint should not be considered as the method to place the mandibular condyles into the most anterior superior part of glenoid fossae, known as centric relation.

Having compared the average changes in the values of different joint spaces and the average changes in condylar ratio between the examined groups, it has been found that only changes in the dimension of superior joint spaces were significantly higher compared to the changes that occurred in control group throughout the period of more than one year. Other changes regarding the remaining joint spaces, as well as changes in the values of condylar ratio between the examined groups were statistically insignificant.

We have found three statistically significant positive correlations between the changes in the dimension of the superior and anterior joint spaces (r = 0.483; *p* < 0.001), superior and posterior joint spaces (r = 0.503; *p* < 0.001), medial and posterior joint spaces (r = 0.291; *p* = 0.009). These correlations are related to the anatomy of TMJs, namely the morphology of glenoid fossae and rotation of condylar heads within the glenoid fossae [23]. Whenever the mandibular condyle goes down, the superior joint space increases. At the same time, the distances between the mandibular condyle and glenoid fossae’ slopes (both the anterior and posterior ones) also increase. To understand the correlation between medial and posterior joint spaces, it must be remembered that condylar heads are not perpendicular to the sagittal plane, but they are slightly rotated toward the foramen magnum [23]. Therefore, with the increase of the medial joint space, also the increase in the dimension of posterior joint space is observed.

Having performed the multivariate logistic regression analysis, there have been found two independent predictors of obtaining anterior condylar position within the glenoid fossa (C_on_R_at_ > 12%) after the end of the treatment in patients diagnosed with TMD. These were C_on_R_at_ before treatment of 0.0% or more, and anterior joint space dimension before treatment less than 2.1 mm. These values describe the clinical situation, in which before the onset of the treatment, the mandibular condyle is positioned in the center of glenoid fossa or anteriorly to the center of glenoid fossa. Therefore, condyles posteriorly displaced within the glenoid fossae before the onset of the treatment cannot be expected to predictably move forward within the glenoid fossa to its anterior part throughout the treatment combining occlusal splint therapy and physiotherapy.

The number of studies based on CBCT images, which assessed the changes in the condylar position within the glenoid fossa after the treatment with occlusal splints is very limited. Ramachandran et al. [24] analyzed the effects of the Kois Deprogrammer worn for three to four weeks with subsequent occlusal equilibration on condylar position in patients diagnosed with TMD. The authors did not notice any statistically significant changes in the mean size of posterior, superior, and medial joint spaces in both TMJs, nor in the mean size of anterior joint space in right TMJs. Although the authors found that mean size of anterior joint space in left TMJs was significantly reduced after the end of the treatment, it should be noted that the p-value was at the verge of statistical significance (*p* = 0.04). Moreover, the authors also noticed that the percentage changes in condylar displacement were statistically insignificant in both joints. The vast majority of those results stay in agreement with our observations. 

Filho et al. [25] analyzed the changes in condylar position in 22 patients diagnosed with TMD who had been prescribed interocclusal stabilization splints to be worn day and night for 90 days (except for eating). The authors performed CBCT examination three times: before the onset of the study, at the end of occlusal splint therapy, and 90 days after the occlusal splint therapy had been completed. Filho et al. [25] noticed that after the end of occlusal splint therapy, the mean value of the superior and posterior joint spaces significantly increased, and the mean value of anterior joint space remained nearly unchanged comparing to the results obtained before the onset of the treatment. The major cause of the differences between the results presented by Filho et al. [25] and the results obtained in our study is the fact that Filho et al. [25] took the second CBCT image in patients who had in their mouth occlusal splints. In our study, the second CBCT image was taken in patients without occlusal splints in their mouths. Therefore, the measurements presented by Filho et al. [25] at the end of occlusal splint therapy may not reflect the exact dimensions of joint spaces, because occlusal splints, even the thinnest ones, increase the vertical dimension. Moreover, occlusal splints may cause rotation of cranium, which also affects the mean size of joint spaces.

Neither Ramachandran et al. [24] nor Filho et al. [25] compared the results obtained in study group to the control group, including healthy volunteers. Both of the studies are missing the control groups.

There can be listed a few limitations to our study. Firstly, the number of participants included into the study is limited. Further studies, especially prospective and multicenter, based on larger groups of patients would be beneficial. Secondly, although we included only adult patients into the study, the age range is wide. Thirdly, the study was based on the CBCT images with the moderate FOV, namely 8 × 5 cm. Larger FOV would allow to perform additional measurements, including position of mandibular condyles interdependently. 

## 5. Conclusions

Occlusal splint therapy and physiotherapy combined together do not change significantly the dimension of anterior, superior, posterior, and medial joint spaces. Moreover, they do not place the mandibular condyles into the centric relation, known as the most anterior superior part of glenoid fossae. In the majority of cases, condyles after the end of occlusal splint therapy are not in centric relation. Treatment of patients with TMD should not aim at gnathological concept of placing the mandibular condyles into centric relation, because centric relation appears not to be mandatory to achieve successful results in patients with TMD.

## Figures and Tables

**Table 1:** Inclusion and exclusion criteria for the study group.

Criteria	List of Specific Criteria
Inclusion criteria	− Diagnosis of TMD on the basis of DC/TMD criteria − Age between 18 and 65 years old − Willingness to participate in the study
Exclusion criteria	− Age below 18 and above 65 years old − History of traumas in the area of head and neck − History of previous orthodontic treatment − Rheumatological diseases − Oncological diseases − Pregnancy − Patients who did not agree to take part in the study

TMJ—temporomandibular joint; TMD—temporomandibular joint disorders.

**Table 2:** List of points and lines used to analyze the position of mandibular condyle within the glenoid fossa.

Points and Lines	Description
MP	Medial pole—the most prominent point on the medial pole of mandibular condyle
LP	Lateral pole—the most prominent point on the lateral pole of mandibular condyle
RGF	Roof of glenoid fossa—the most superior point of the glenoid fossa
MGF	Medial wall of glenoid fossa—point in the axial view, which is the intersection of coronal axis line and medial wall of glenoid fossa
AAL	Auxiliary anterior line—auxiliary line from RGF to the most anterior point on the mandibular condyle
APL	Auxiliary posterior line—auxiliary line from RGF to the most posterior point on the mandibular condyle
AJS	Anterior joint space—the distance between the most anterior point on the mandibular condyle and the posterior slope of articular eminence, measured across the line, which is perpendicular to AAL
SJS	Superior joint space—the distance between RGF and the most superior point on the mandibular condyle
PJS	Posterior joint space—the distance between the most posterior point on the mandibular condyle and the posterior wall of glenoid fossa, measured across the line, which is perpendicular to APL
MJS	Medial joint space—the distance between MP and MGF

**Table 3:** Interpretation of the Pullinger and Hollender’s formula on the basis of the literature [20].

Condylar Ratio	Interpretation
0 ± 12%	Concentric position of the mandibular condyle within the glenoid fossa
Less than −12%	Posterior position of the mandibular condyle within the glenoid fossa
More than 12%	Anterior position of the mandibular condyle within the glenoid fossa

**Table 4:** Comparison of age and sex between the examined groups.

Variables	Study Group (*n* = 40)	Control Group (*n* = 15)	*p*-Value
**Age** (years)			
av. (SD)	28.4 (10.2)	31.3 (12.9)	0.217 ^a^
range	18–61	18–58	
median (Q_1_;Q_3_)	27 (21;34)	32 (21;38)	
**Sex**			
Female, *n* (%)	32 (80.0%)	12 (80.0%)	1.000 ^b^
Male, *n* (%)	8 (20.0%)	3 (20.0%)	

^a^ U Mann–Whitney test; ^b^ chi-square test; av.—average; SD—standard deviation; Q1—lower quartile; Q3—upper quartile; AJS—anterior joint space; PJS—posterior joint space; SJS—superior joint space; MJS—medial joint space.

**Table 5:** The average values of maximum mouth opening and pain scores before and after the end of the treatment.

Measurement	Before Treatment (*n* = 40)	After Treatment (*n* = 40)	*p*-Value
**Maximum mouth opening** (mm)			
av. (SD)	42.15 (6.38)	48.15 (5.74)	0.0011 ^a^
range	30–57	31.5–61	
median	43.5	48.75	
**Pain score**			
av. (SD)	1.8 (0.8)	0.05 (0.2)	< 0.0001 ^a^
range	1–3	0–1	
median	1	0	

^a^ Student *t* test; av.—average; SD—standard deviation.

**Table 6:** The average values of different joint spaces and condylar ratio during initial and second examination in the control group.

Measurement	Initial Examination (*n* = 30)	Second Examination (*n* = 30)	*p*-Value
**AJS** (mm)			
av. (SD)	2.50 (0.81)	2.50 (0.80)	0.801 ^a^
range	1.1–4.6	1.1–4.6	
median (Q_1_;Q_3_)	2.4 (1.9;2.8)	2.4 (1.9;2.8)	
**PJS** (mm)			
av. (SD)	2.03 (0.59)	2.03 (0.59)	0.645 ^a^
range	0.8–3.9	0.8–3.8	
median (Q_1_;Q_3_)	2.0 (1.7;2.3)	2.0 (1.7;2.4)	
**SJS** (mm)			
av. (SD)	3.30 (0.77)	3.29 (0.74)	0.380 ^a^
range	2.1–4.7	2.1–4.6	
median (Q_1_;Q_3_)	3.3 (2.5;4.1)	3.3 (2.6;3.9)	
**MJS** (mm)			
av. (SD)	4.28 (1.20)	4.28 (1.20)	0.873 ^a^
range	2.1–6.7	2.1–6.7	
median (Q_1_;Q_3_)	4.2 (3.5;5.2)	4.2 (3.4;5.1)	
**Condylar ratio** (%)			
av. (SD)	−9.4 (23.2)	−9.2 (23.1)	0.696 ^a^
range	−61.0–47.0	−60.0–43.0	
median (Q_1_;Q_3_)	−4.0 (−24.0;2.0)	−2.0 (−28.0;2.0)	
**Condylar position**			
Posterior (C_on_R_at_ < −12%)	11 (36.7%)	11 (36.7%)	1.000 ^b^
Central (−12% ≤ C_on_R_at_ ≤ 12%)	16 (53.3%)	16 (53.3%)	
Anterior (C_on_R_at_ >12%)	3 (10.0%)	3 (10.0%)	

^a^ Student *t* test; ^b^ chi-square test; av.—average; C_on_R_at_—condylar ratio; SD—standard deviation; Q1—lower quartile; Q3—upper quartile; AJS—anterior joint space; PJS—posterior joint space; SJS—superior joint space; MJS—medial joint space.

**Table 7:** The average values of different joint spaces and condylar ratio before and after the end of the treatment in the study group.

Measurement	Before Treatment (*n* = 80)	After Treatment (*n* = 80)	*p*-Value
**AJS** (mm)			
av. (SD)	2.9 (1.28)	2.89 (1.29)	0.989 ^a^
range	0.8–7.5	1.1–7.8	
median (Q_1_;Q_3_)	2.7 (2.0;3.3)	2.7 (2.2;3.2)	
**PJS** (mm)			
av. (SD)	2.18 (0.74)	2.38 (0.93)	0.343 ^a^
range	0.8–5.1	1.1–4.9	
median (Q_1_;Q_3_)	2.1 (1.7;2.6)	2.2 (1.7;2.9)	
**SJS** (mm)			
av. (SD)	3.18 (1.09)	3.44 (1.05)	0.108 ^b^
range	1.2–6.3	1.5–6.3	
median (Q_1_;Q_3_)	3.2 (2.4;3.8)	3.4 (2.6;4.2)	
**MJS** (mm)			
av. (SD)	4.41 (1.77)	4.66 (1.7)	0.361 ^b^
range	1.2–8.7	0.7–8.3	
median (Q_1_;Q_3_)	4.5 (3.1;5.7)	4.9 (3.4;5.7)	
**Condylar ratio** (%)			
av. (SD)	−12.5 (23.8)	−8.8 (25.6)	0.025 ^b^
range	−63–50	−64.2–57.9	
median (Q_1_;Q_3_)	−13 (−30.1;0.7)	−12.1 (−25.6;7.2)	
**Condylar position**			
Posterior (C_on_R_at_ < −12%)	42 (52.5%)	39 (48.75%)	0.346 ^c^
Central (−12% ≤ C_on_R_at_ ≤ 12%)	27 (33.8%)	23 (28.75%)	
Anterior (C_on_R_at_ > 12%)	11 (13.7%)	18 (22.5%)	

^a^ U Mann–Whitney test; ^b^ Student *t* test; ^c^ chi-square test; av.—average; C_on_R_at_—condylar ratio; SD—standard deviation; Q1—lower quartile; Q3—upper quartile; AJS—anterior joint space; PJS—posterior joint space; SJS—superior joint space; MJS—medial joint space.

**Table 8:** The average changes in the values of different joint spaces after the end of the treatment in right and left TMJs in the study group.

Measurement	Right TMJ (*n* = 40)	Left TMJ (*n* = 40)	*p*-Value
Δ**AJS** (mm)			
av. (SD)	0.06 (0.51)	−0.08 (0.62)	0.224 ^a^
range	−1.1–1.4	−1.8–1.5	
median (Q_1_;Q_3_)	0.0 (−0.3;0.3)	0.0 (−0.3;0.3)	
Δ**PJS** (mm)			
av. (SD)	0.19 (0.6)	0.23 (0.7)	0.694 ^a^
range	−1.2–1.4	−5.0–2.4	
median (Q_1_;Q_3_)	0.2 (−0.3;0.7)	0.0 (−0.2;0.4)	
Δ**SJS** (mm)			
av. (SD)	0.29 (0.58)	0.23 (0.65)	0.635 ^a^
range	−0.7–1.8	−1.5–2.0	
median (Q_1_;Q_3_)	0.3 (0;0.7)	0.3 (−0.1;0.5)	
Δ**MJS** (mm)			
av. (SD)	0.2 (0.93)	0.3 (1.22)	0.692 ^a^
range	−1.2–3.1	−3.9–2.8	
median (Q_1_;Q_3_)	0.1 (−0.5;0.8)	0.2 (−0.2;0.8)	

^a^ Student *t*-test; av.—average; SD—standard deviation; Q1—lower quartile; Q3—upper quartile; AJS—anterior joint space; PJS—posterior joint space; SJS—superior joint space; MJS—medial joint space, TMJ—temporomandibular joint, Δ—difference between the values after and before the treatment (i.e., ΔAJS = AJS _after treatment_ − AJS _before treatment_).

**Table 9:** The results of the treatment assessed on the basis of the of the condylar ratio changes in the study group.

Effect of the Treatment	Right TMJ (*n* = 40)	Left TMJ (*n* = 40)	*p*-Value
Positive ΔC_on_R_at_ ≥ 5%	18 (45%)	19 (47.5%)	0.727 ^a^
Neutral −5% < ΔC_on_R_at_ < 5%	10 (25%)	12 (30%)	
Negative ΔC_on_R_at_ ≤ −5%	12 (30%)	9 (22.5%)	

^a^ Chi-square test; C_on_R_at_—condylar ratio; TMJ—temporomandibular joint, Δ—difference between the values after and before the treatment (ΔC_on_R_at_ = C_on_R_at after treatment_ − C_on_R_at before treatment_).

**Table 10:** Comparison of the average changes in the values of different joint spaces and the average changes in condylar ratio between the examined groups.

Measurement	Study Group (*n* = 80)	Control Group (*n* = 30)	*p*-Value
Δ**AJS** (mm)			
av. (SD)	−0.01 (0.57)	0.00 (0.07)	0.924 ^a^
range	−1.2–2.4	−0.1–0.2	
median (Q_1_;Q_3_)	0.2 (−0.2;0.6)	0.0 (0.0;0.0)	
Δ**PJS** (mm)			
av. (SD)	0.21 (0.65)	0.01 (0.08)	0.097 ^a^
range	−1.2–2.4	−0.1–0.3	
median (Q_1_;Q_3_)	0.2 (−0.2;0.6)	0.0 (0.0;0.0)	
Δ**SJS** (mm)			
av. (SD)	0.26 (0.61)	−0.01 (0.08)	0.018 ^a^
range	−1.5–2.0	−0.2–0.2	
median (Q_1_;Q_3_)	0.3 (0.0;0.6)	0.0 (−0.2;0.2)	
Δ**MJS** (mm)			
av. (SD)	0.25 (1.08)	0.0 (0.11)	0.210 ^a^
range	−3.9–3.1	−0.3–0.3	
median (Q_1_;Q_3_)	0.1 (−0.4;0.8)	0.0 (0.0;0.1)	
Δ**Condylar ratio** (%)			
av. (SD)	3.7 (14.3)	−0.2 (2.8)	
range	−29.0–40.0	−6.0–8.0	0.143 ^a^
median (Q_1_;Q_3_)	4.0 (−6.0;13.0)	0.0 (0.0;1.0)	
**Treatment effect**			
Positive ΔC_on_R_at_ ≥ 5%, *n* (%)	37 (46.25%)	0 (0.0%)	
Neutral −5% < ΔC_on_R_at_ < 5%, *n* (%)	22 (27.5%)	30 (100.0%)	<0.001 ^b^
Negative ΔC_on_R_at_ ≤ −5%, *n* (%)	21 (26.25%)	0 (0.0%)	

^a^ Student *t*-test; ^b^ chi-square test; av.—average; SD—standard deviation; Q1—lower quartile; Q3—upper quartile; AJS—anterior joint space; PJS—posterior joint space; SJS—superior joint space; MJS—medial joint space, TMJ—temporomandibular joint, Δ—difference between the values after and before the treatment (i.e., ΔC_on_R_at_ = C_on_R_at after treatment_ − C_on_R_at before treatment_).

**Table 11:** The results of the ROC curves analysis.

Parameter	Cutoff Value	Sensitivity	Specificity	AUC	95% CI for AUC
C_on_R_at before treatment_	0%	88.9%	83.9%	0.91	(0.83;0.98)
AJS _before treatment_	<2.1 mm	86.7%	77.8%	0.85	(0.73;0.97)
PJS _before treatment_	>2.4 mm	72.2%	80.0%	0.77	(0.66;0.89)
SJS _before treatment_	>2.6 mm	88.9%	35.5%	0.65	(0.52;0.78)
MJS _before treatment_	>3.5 mm	72.2%	40.0%	0.53	(0.38;0.68)

AJS—anterior joint space; AUC—area under the curve; CI—confidence interval; C_on_R_at_—condylar ratio; MJS—medial joint space; PJS—posterior joint space; SJS—superior joint space.

**Table 12:** Predictive factors for obtaining anterior condylar position after the end of the treatment.

Predictive Factor	C_on_R_at_ after Treatment		*p*-Value	OR (95% CI)
	C_on_R_at_ ≥ 12% *n* (%)	C_on_R_at_ < 12% *n* (%)		
C_on_R_at before treatment_				
C_on_R_at_ ≥ 0.0%	16 (88.9%)	10 (16.1%)	<0.001	41.6 (8.25;210)
C_on_R_at_ < 0.0%	2 (11.1%)	52 (83.9%)		
AJS _before treatment_				
AJS < 2.1 mm	14 (77.8%)	10 (16.1%)	<0.001	18.2 (4.95;66.9)
AJS ≥ 2.1 mm	4 (22.2%)	52 (83.9%)		
PJS _before treatment_				
>2.4 mm	14 (77.8%)	24 (38.7%)	0.006	5.54 (1.63;18.8)
≤2.4 mm	4 (22.2%)	38 (61.3%)		
SJS _before treatment_				
>2.6 mm	16 (88.9%)	40 (64.5%)	0.077	4.40 (0.93;20.9)
≤2.6 mm	2 (11.1)	22 (35.5%)		
MJS _before treatment_				
>3.5 mm	13 (72.2%)	38 (61.3%)	0.568	1.64 (0.52;5.19)
≤3.5 mm	5 (27.8%)	24 (38.7%)		

AJS—anterior joint space; CI—confidence interval; C_on_R_at_—condylar ratio; MJS—medial joint space; OR—odds ratio; PJS—posterior joint space; SJS—superior joint space.

**Table 13:** The results of the univariate and multivariate logistic regression analyses.

Predictive Factor	Univariate Logistic Regression		Multivariate Logistic Regression		
	Beta	*p*-Value	Beta	*p*-Value	OR (95% CI)
C_on_R_at before treatment_ ≥ 0.0%	3.728	<0.001	2.974	0.001	19.6 (3.46;111)
AJS _before treatment_ < 2.1 mm	2.901	<0.001	1.673	0.033	5.33 (1.14;24.9)
PJS _before treatment_ > 2.4 mm	1.712	0.006	–	>0.05	–
SJS _before treatment_ > 2.6 mm	1.085	0.064	–	>0.05	–
MJS _before treatment_ > 3.5 mm	0.496	0.398	–	>0.05	–

AJS—anterior joint space; AUC—area under the curve; CI—confidence interval; C_on_R_at_—condylar ratio; MJS—medial joint space; OR—odds ratio; PJS—posterior joint space; SJS—superior joint space.

## Data Availability

The data underlying this article are available in the article.
